# Mouse Strain Affects Behavioral and Neuroendocrine Stress Responses Following Administration of Probiotic *Lactobacillus rhamnosus* JB-1 or Traditional Antidepressant Fluoxetine

**DOI:** 10.3389/fnins.2018.00294

**Published:** 2018-05-08

**Authors:** Karen-Anne McVey Neufeld, Sebastian Kay, John Bienenstock

**Affiliations:** ^1^Department of Pathology and Molecular Medicine, McMaster University, Hamilton, ON, Canada; ^2^Brain-Body Institute, St. Joseph's Healthcare at McMaster University, Hamilton, ON, Canada

**Keywords:** microbiota-gut-brain axis, probiotic, antidepressant, mouse strain, stress reactivity

## Abstract

Currently, there is keen interest in the development of alternative therapies in the treatment of depression. Given the explosion of research focused on the microbiota-gut-brain axis, consideration has turned to the potential of certain probiotics to improve patient outcomes for those suffering from mood disorders. Here we examine the abilities of a known antidepressant, fluoxetine, and the probiotic *Lactobacillus rhamnosus* JB-1™, to attenuate responses to two established criteria for depressive-like behavior in animal models, the tail suspension test (TST) and the corticosterone response to an acute restraint stressor. We examine two different strains of mice known to differ in the extent to which they express both anxiety-like behavior and measures of despair—BALB/c and Swiss Webster—with respectively high and normal behavioral phenotypes for each. While adult male BALB/c mice responded with increased antidepressive-like behavior to both fluoxetine and *L. rhamnosus* JB-1 in both the TST and the corticosterone stress response, SW mice did not respond to either treatment as compared to controls. These findings highlight the importance of investigating putative antidepressants in mouse strains known to express face validity for some markers of depression. Clinical studies examining the activity of *L. rhamnosus* JB-1 in patients suffering from mood disorders are warranted, as well as further pre-clinical work examining how interactions between host genotype and intestinal microbial alterations may impact behavioral responses. This study adds to the literature supporting the possibility that modifying the intestinal microbiota via probiotics represents a promising potential therapeutic breakthrough in the treatment of psychiatric disease.

## Introduction

Currently there is intense interest in the development of alternative therapies for the treatment of psychiatric illness. With the explosion of research activity focused on the microbiota-gut-brain axis, attention has turned to the potential of microbial-based therapies in disorders of the central nervous system (CNS), especially with regards to mood disorders. According to the World Health Organization, more than 300 million people suffer from depression worldwide, and it is the leading cause of disability (WHO, 2017). Traditional antidepressants are considered effective in only 20–30% of the population suffering from depression (Davis et al., 1993; Walsh et al., 2002; Arroll et al., 2005), and probiotics are an intriguing possible treatment currently being explored (Logan and Katzman, 2005; Dinan et al., 2013).

The microbiota-gut-brain axis is a bi-directional axis of communication that is mediated by the endocrine, immune and neural systems (Berthoud, 2008; El Aidy et al., 2014; Mayer et al., 2015). Initial interest in this axis was largely driven by attempts to understand the pathogenesis of irritable bowel syndrome (IBS), a functional bowel disorder in patients that is frequently comorbid with mood disorders such as anxiety and depression (Whitehead et al., 2002). Probiotics are currently used in the successful treatment of IBS symptoms and there is question as to whether their efficacy is related to improvements in bowel and/or brain function (Ford et al., 2014; Pinto-Sanchez et al., 2017; Ait-Belgnaoui et al., 2018). Indeed, pre-clinical work in mice has demonstrated the anxiolytic effects of certain beneficial bacteria, as well as showing anti-depressive effects and improved cognition following treatment (Bravo et al., 2011; Savignac et al., 2014, 2015; Marin et al., 2017). In spite of the promising preclinical findings with respect to CNS change following probiotic treatment, some uncertainty with respect to translation to humans remains. A recent study conducted in healthy volunteers found that *Lactobacillus rhamnosus* JB-1™ resulted in no improvement to stress-related measures or cognition scores after 4 weeks of treatment (Kelly et al., 2017). However, given that treatment was provided to an already healthy volunteer population as opposed to a more vulnerable clinical cohort, we sought to further examine this in mice.

BALB/c mice are genetically inbred mice with known increases in anxiety-like behavior as compared to other mouse strains, while Swiss Webster (SW) mice are considered to be mid-range or normal with respect to predisposition to anxiety-like behavior (Crawley, 2008). In addition, BALB/c mice are considered one of the most consistently responsive strains in behavioral tests of despair (Jacobson and Cryan, 2007) and are thus often preferentially selected as animal models of the mood disorders anxiety and depression, as they arguably better reflect the core features observed in these human patients (Jacobson and Cryan, 2007; Sartori et al., 2011). In this study we chose to examine the responses to treatment with either a known antidepressant fluoxetine or probiotic JB-1 in both SW and BALB/c mice. We measured both a behavioral and physiological response to treatment consistent with the clinical presentation of depression in humans, depressive-like behavior in the tail suspension test (TST) and stress reactivity via measures of plasma corticosterone following a stressor. We sought to examine if responses to both a known and putative antidepressant differed in mouse strains selected on the basis of differing vulnerability to anxiety-like and depressive-like behavior.

## Materials and methods

### Animals

Six- to eight-week old male BALB/c (*n* = 46, average weight 26 g) and Swiss Webster (SW) (*n* = 36, average weight 35 g) mice were purchased from Charles River (Montreal, QC, Canada) and allowed to habituate to the animal facility for 1 week. Animals were housed 4–5/cage and kept under controlled conditions (21 ± 1°C) on a 12-h light/dark cycle (lights on at 5:00 a.m.) and fed *ad libitum*. All experiments were carried out in accordance with the guidelines of the Canadian Council on Animal Care and were approved by McMaster University's Animal Research Ethics Board.

### Oral treatment

BALB/c and Swiss Webster mice were randomized into *L. rhamnosus* JB-1™ (JB-1), fluoxetine hydrochloride (Sigma Millipore) or plain drinking water groups (*n* = 12–22/group). JB-1 was received as a gift from Alimentary Health, Cork, Ireland and is the same bacterial strain we have previously used in our laboratory (Bravo et al., 2011; Leclercq et al., 2017). JB-1 was delivered via the drinking water at a dose of 10^9^ CFU/day at which it remains viable for more than 12 h. We have previously demonstrated that JB-1 delivered at this dose via the drinking water passes through the defenses of the stomach intact, and can be demonstrated in the fecal pellets of treated mice (Leclercq et al., 2017). Water bottles containing JB-1 were freshly prepared every morning and shaken multiple times throughout the course of the day to ensure adequate mixing of material. Animals were treated with JB-1 for 28 days. Animals randomized to the fluoxetine treated group received drug via the drinking water at a dose of 18 mg/kg for 14 days. Animals randomized to the plain drinking water group were treated similarly, but received no JB-1 or fluoxetine.

### Tail suspension test (TST)

One day following oral treatments, animals were tested for depressive-like behavior with the TST. Mice were removed from the colony room and moved to a behavioral testing room where they were allowed to habituate for 30 min. Following this period, mice were suspended by the tail using laboratory tape measured to 17 cm (Can et al., 2012) from a suspension bar in a position whereby they could not escape or hold on to any surfaces. Two centimeters of tape was affixed to the mouse tail with the remaining 15 cm used for suspension of the mouse. Animals were left suspended for a period of 6 min. Animal behavior was video recorded and scored by a blinded observer for freezing behavior. Freezing was calculated as a percentage of total time. Following behavioral testing mice were returned to the housing room and resumed oral treatments for the duration of the study.

### Acute restraint stress and serial blood collection

Four days after the TST animals were moved to a behavioral testing room at 08:30 and allowed to habituate for 30 min. Following this period, animals were removed from their home cages and gently placed on a table where their tail tip was cut at an angle with a scalpel blade and blood collected via capillary tube (~20 μL/time point) which was decanted into 1.5 mL collection tube containing 5 μL EDTA. The first blood collection was marked T0 and corresponded to baseline collection. Animals were then placed in wire mesh restrainers and singly housed for 30 min (Conrad et al., 2017). Following this, animals were removed from the restrainers and the T30 blood collection occurred. The animal was again placed into a single cage, but now unrestrained. Tail bleeds were thus performed every 30 min from T0 (beginning of restraint) to T120 (animal returned to home cage). All bloods were collected between the hours of 09:00 and 12:00 in order to control for variation in circadian release of corticosterone. Blood was stored on wet ice until spun in a centrifuge and plasma collected. Plasma was stored in a −80°C freezer until an ELISA assay (Enzo) for corticosterone was performed.

### Statistics

Both one-way and two-way ANOVAs were carried out with a Bonferroni correction for *post-hoc* measures. All behavior was scored by a single blinded observer.

## Results

### Feeding *L. rhamnosus* JB-1 or fluoxetine to BALB/c mice reduces depressive-like behavior

**Figure 1:**
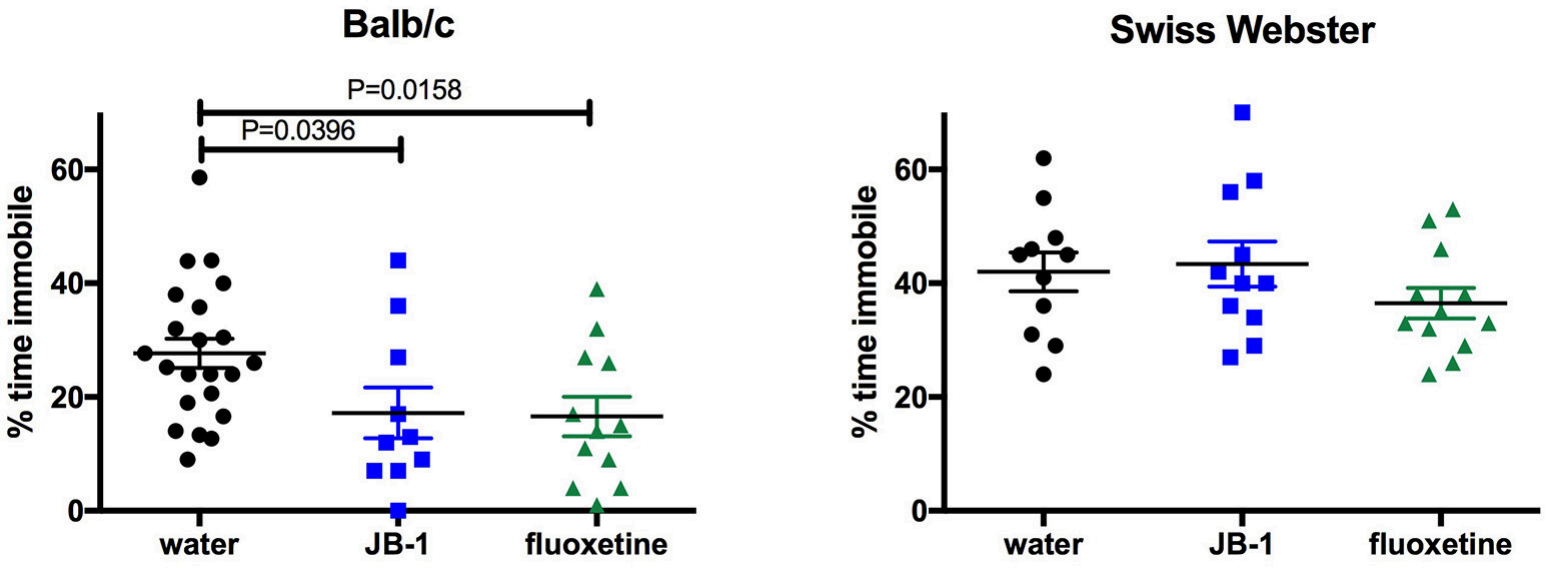
Feeding fluoxetine or JB-1 to BALB/c mice reduces depressive-like behavior. Twenty-eight-day treatment of *Lactobacillus rhamnosus* JB-1 supplied via the drinking water reduced depressive-like behavior in BALB/c mice, but not Swiss Webster mice, as measured by % total time immobile in the tail suspension test. One-way ANOVA with corrected *post-hoc p* = 0.0396, *n* = 11–22/grp. Fourteen day treatment with fluoxetine via the drinking water reduced depressive-like behavior in BALB/c, but not Swiss Webster mice, as measured by % total time immobile. One-way ANOVA with corrected *post-hoc p* = 0.0158, *n* = 11–22/grp.

One-way ANOVA showed a main effect for treatment [*F*_(2, 41)_ = 4.072, *p* = 0.0244] in BALB/c mice and corrected *post-hoc* analyses demonstrated that feeding JB-1 for 28 days in drinking water decreased depressive-like behavior in the TST as compared to control mice (*p* = 0.0396). This was observed by a significant decrease in freezing behavior/time spent immobile in JB-1 fed mice (see Figure 1). Feeding BALB/c mice fluoxetine for 14 days in drinking water similarly decreased depressive-like behavior in the TST compared to water fed controls (*p* = 0.0158, *n* = 12–22/grp; see Figure 1).

One-way ANOVA shows no effect of treatments [*F*_(2, 31)_ = 1.2, *p* = 0.3147, *n* = 12/grp] in SW mice fed JB-1 or fluoxetine in the TST (see Figure 1).

### Feeding *L. rhamnosus* JB-1 or fluoxetine to BALB/c mice attenuates plasma corticosterone and hastens recovery

**Figure 2:**
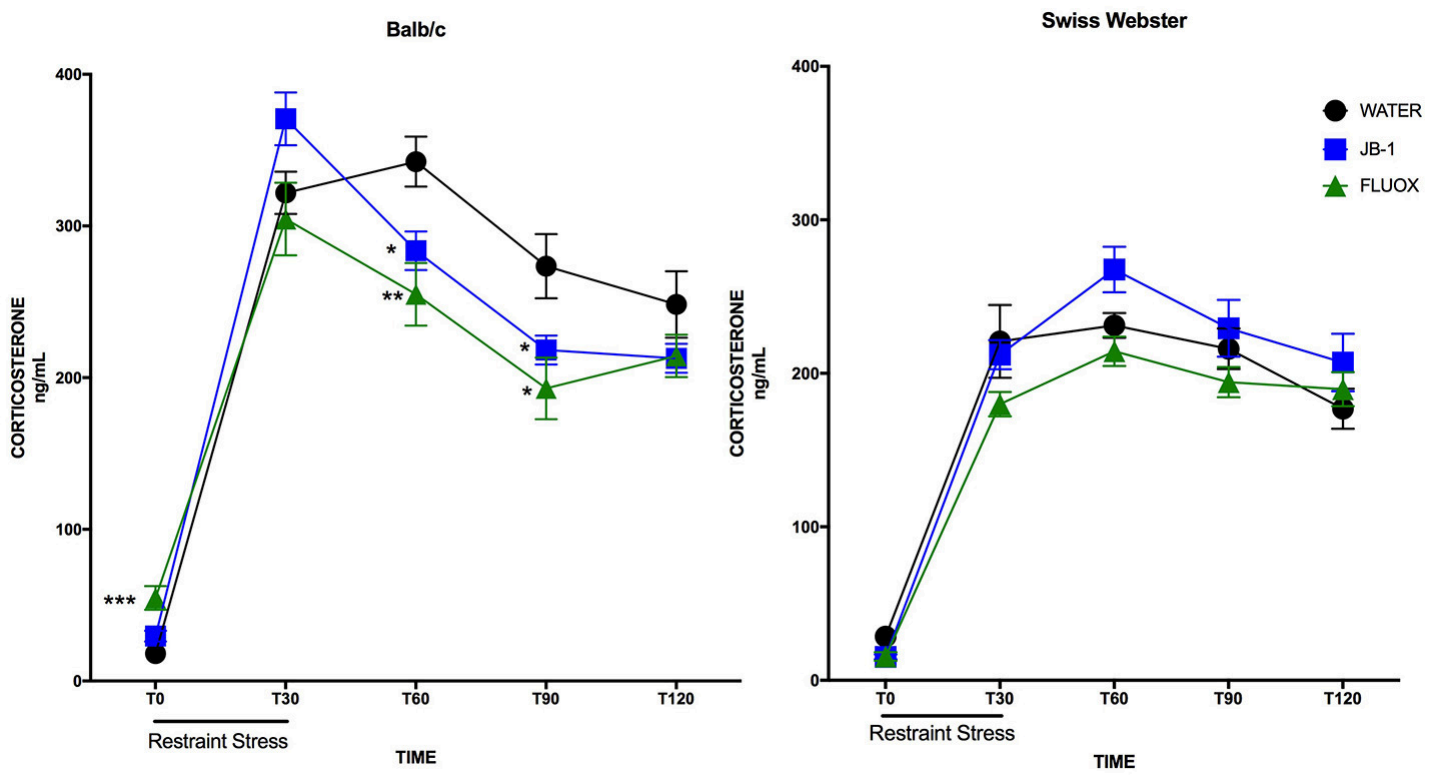
Feeding fluoxetine or JB-1 to BALB/c mice attenuates stress reactivity. Twenty-eight-day treatment of *Lactobacillus rhamnosus* JB-1 supplied via the drinking water reduced plasma corticosterone levels following restraint stress in BALB/c as compared to water fed controls, but did not in Swiss Webster mice. Two-way repeated measures ANOVA with corrected *post-hoc*, T60 *p* = 0.019 and T90 *p* = 0.05, *n* = 12/grp. Fourteen day treatment with fluoxetine via the drinking water reduced plasma corticosterone levels following restraint stress in BALB/c as compared to water fed controls, but did not in Swiss Webster mice. Two-way repeated measures ANOVA with corrected *post-hoc*, T60 *p* = 0.0072, T90 *p* = 0.023, *n* = 12/grp.

In BALB/c mice, a two-way repeated measures ANOVA showed a main effect for time [*F*_(4, 132)_ = 185.8, *p* = 0.0001], and a significant interaction [*F*_(8, 132)_ = 4.83, *p* = 0.0001] but no main effect for treatment [*F*_(2, 33)_ = 2.486, *p* = 0.0987] in plasma corticosterone levels after an acute restraint stress. Corrected *post-hoc* analyses demonstrated a significant decrease in plasma corticosterone levels in JB-1 fed mice vs. control at T60 (^*^p = 0.019), and at T90 (^*^p = 0.05) (see Figure 2). Fluoxetine treatment led to a similar decrease in plasma corticosterone compared to controls at T60 (^**^p = 0.0072) and T90 (^*^p = 0.023). In addition, fluoxetine feeding led to a significant increase in basal plasma corticosterone compared to controls, T0 (^***^p = 0.0006) (*n* = 12/grp; see Figure 2).

In Swiss Webster mice, a two-way repeated measures ANOVA showed a main effect for time [*F*_(4, 132)_ = 127.3, *p* = 0.0001], but no effect of treatment [*F*_(2, 33)_ = 2.662, *p* = 0.0848] or interaction [*F*_(8, 132)_ = 1.208, *p* = 0.2989]. *Post-hoc* analyses showed no significant differences in either treatment group as compared to controls at individual time points (*n* = 12/grp; see Figure 2).

## Discussion

The purpose of this study was to examine both a behavioral and physiological response to treatment with a well-known antidepressant, the selective serotonin reuptake inhibitor fluoxetine, and the probiotic JB-1 in mice established to have both high (BALB/c) and normal (SW) anxiety-like traits as well as the propensity to score high (BALB/c) and normal (SW) on measures of despair. Here we observe the antidepressive-like effects of both probiotic JB-1 and fluoxetine in adult male BALB/c mice but not SW mice. We also observe alterations in hypothalamic-pituitary-adrenal axis activity in BALB/c animals following both the probiotic and antidepressant treatments, with mice demonstrating reduced plasma corticosterone following a 30 min restraint stress in both treatment groups as compared to control animals. Again, this reduction in stress reactivity following treatment with probiotic or antidepressant was not observed in SW mice. Interestingly, the attenuating effects of both JB-1 and fluoxetine in BALB/c mice on corticosterone levels following the restraint stress are obvious during the “recovery” phase of stress responsivity (i.e., 30–60 min following removal from the restrainer). Both treatments resulted in a faster recovery toward basal corticosterone levels which is generally viewed as positive as it potentially protects against detrimental effects due to sustained exposure to high corticosterone (Szuran et al., 2000). Recovery or termination of the stress response occurs via negative feedback control and activation of the glucocorticoid receptors (Jacobson and Sapolsky, 1991). While we did not characterize glucocorticoid receptor expression in the brains of our animals, this would be an interesting follow up to the present study. Our findings indicate a strain-specific effect in mice, in that the BALB/c respond to either probiotic or antidepressant in both behavioral and physiological measures of depressive-like behavior and stress reactivity, while the SW mice do not.

Recently, certain bacterial strains have been touted for their potential to act as “psychobiotics”—and indeed interest is high given the lower response rates reported for traditional antidepressants currently available (Dinan et al., 2013). JB-1 is a promising bacterial strain pre-clinically, in that it has displayed the ability to modify animal behavior across a panel of well-validated behavioral assays (Bravo et al., 2011) as well as demonstrated anti-inflammatory effects (Karimi et al., 2009, 2012; Forsythe et al., 2012).

The selection of the TST was based on its established validity for measuring animal behavior most consistent with depression in humans (Steru et al., 1985; Cryan et al., 2005). While there are other behavioral tests commonly used to measure depressive-like behavior or despair in rodents, such as the forced swim test (FST) or sucrose preference test, both of these were rejected in the current study. The first as we have previously demonstrated reduced depressive-like behavior in BALB/c mice treated with JB-1 in the FST (Bravo et al., 2011), and we were seeking an alternate measure in order to further strengthen our previous observations. Sucrose preference test was rejected on the basis of potential confounders in using a sweetened sugar solution in probiotic studies. Not only are we unsure if the oral probiotic 28 d treatment the JB-1 group was subjected to is in and of itself sweet (and thus might have “trained” the mice to show preference for a sweeter solution), but it is also possible that the sucrose could differentially alter the microbiome of mice after the first behavioral test. For this reason we selected the TST as the best and most reliable test of depressive-like behavior for the current study. The corticosterone response to stress was selected as a marker of an antidepressant-like physiological measure as HPA axis alterations have been consistently reported in patients suffering with mood disorder for the last 30 years (Varghese and Brown, 2001), and indeed dysregulation of the HPA axis is one of the most replicated findings in a subset of patients suffering from depression (Varghese and Brown, 2001; Keller et al., 2017).

BALB/c mice are recognized as being “anxiety-prone” or “highly anxious” compared to other strains of mice (Sartori et al., 2011; Savignac et al., 2011b), as well as to be most consistently responsive in behavioral measures of despair (Jacobson and Cryan, 2007). BALB/c mice demonstrate increased anxiety as measured in the light/dark test as compared to both SW and C57BL/6 (Griebel et al., 2000; O'Mahony et al., 2010), with associated changes in c-Fos protein expression in the prefrontal cortex (O'Mahony et al., 2010). BALB/c mice show an increased anxiety-like behavior in the step-down test compared to NIH Swiss, and provokingly, this phenotype of BALB/c mice can be transferred to the NIH Swiss mice via fecal microbial transfer (Bercik et al., 2011). Previous work has also demonstrated the BALB/c susceptibility to both acute and chronic social stress, with mice showing increased plasma corticosterone and cytokine expression as well as damage to colon tissue following both 1 day and 6 day exposure to a dominant aggressive mouse (Savignac et al., 2011b). Indeed in a follow up study by the same group, BALB/c mice were shown to be susceptible to a short duration of social defeat, while C57BL/6 mice exposed to the same paradigm did not show subsequent behavioral impairment (Savignac et al., 2011a). Predisposition to anxiety and sensitivity to stress are thus essential variables to consider in the selection of appropriate mouse strains intended to model psychiatric illness, and indeed even in the human population it is recognized that certain individuals are more genetically susceptible to the negative results of stress while others are more resilient (Feder et al., 2009).

The importance of considering variability in mouse strains with respect to anxiety-like behavior and stress responsivity may be especially true when designing studies to screen putative antidepressants (Belzung, 2014). Changes to behavior and physiological responses to stressors may not be observable in subjects considered normal with respect to anxiety traits. In a recent study examining the effects of chronic oral treatment of SW mice with the antidepressants buproprion, desipramine, and fluoxetine, all failed to result in antidepressant-like behavior as measured in the FST (Suman et al., 2017). Indeed in another study measuring responses to chronic fluoxetine in a variety of mouse strains, a paradoxical increase in immobility in the TST was observed in the SW mice (Miller et al., 2008). Further, in a meta-analysis examining the response of healthy human subjects to antidepressants, no differences were found in mood or anxiety symptoms between those receiving either antidepressants or placebo (Serretti et al., 2010). In fact, this is not a novel finding as the literature frequently shows healthy human volunteers do not respond to antidepressant treatment (Barr et al., 1997; Gelfin et al., 1998). Interestingly, this may in fact explain some of the results of a recent study examining the potential anti-anxiety and antidepressive effects of JB-1 in human volunteers. Kelly et al. (2017) reported that JB-1 was ineffective in improving scores of depression and anxiety in healthy human volunteers, but given the absence of clinical depression in the participants at the outset of treatment, it is unclear what improvements in scoring could be observed. Indeed it will be interesting to see if different results are obtained if the study is repeated in human subjects given JB-1 with a diagnosis of anxiety or depressive disorders. Of note, a separate study has shown that treatment with the probiotic *Bifidobacterium longum* results in improvements in depression scores in the healthy human population, indicating that there may be differences in mode of action depending on bacterial strain (Allen et al., 2016).

The findings of this paper reflect the necessity of careful selection of mouse strain in study design when attempting to model certain disease states pre-clinically. In this case, a mouse strain with established behavioral phenotype (“anxiety-prone”) that more closely exhibits the face validity of the human population suffering from mood disorders was needed in order to observe treatment effects. We argue here that the BALB/c mouse strain thus shows more face validity compared to the SW mouse in modeling human anxiety and depressive disorders, and indeed only observe the known antidepressant effects of both fluoxetine and the pharmabiotic JB-1 in the BALB/c animals. The argument for improved animal modeling of psychiatric disease was laid out clearly by Belzung and Lemoine (2011), who refined Willner's criteria for animal models in psychiatric illness (Willner, 1984). The authors argue that homological validity of an animal model requires that appropriate animal strains be used based on genetic vulnerability and the particular psychiatric symptom/illness in question.

Here we highlight the importance of a carefully selected animal model in the screening of potential psychobiotics aimed at the treatment of depression and stress-related psychiatric disorders. Further work examining the effects of JB-1 in the clinically depressed patient population is required and could aid in the discovery of microbial-based therapies for the treatment of psychiatric disease.

## Author contributions

KAMN designed the experiment, conducted animal behavioral experiments, restraint stress/blood collection, data analyses, and wrote the manuscript; SK conducted laboratory assays and analyses; JB designed the experiment and provided critical advice.

### Conflict of interest statement

The authors declare that the research was conducted in the absence of any commercial or financial relationships that could be construed as a potential conflict of interest.

## Abbreviations

CNS: central nervous system
IBS: irritable bowel syndrome
SW: Swiss Webster
TST: tail suspension test.
